# Association between preoperative platelet and 30-day postoperative mortality of adult patients undergoing craniotomy for brain tumors: data from the American College of Surgeons National Surgical Quality Improvement Program database

**DOI:** 10.1186/s12883-022-03005-5

**Published:** 2022-12-09

**Authors:** Yufei Liu, Haofei Hu, Zongyang Li, Jihu Yang, Xiejun Zhang, Lei Chen, Fanfan Chen, Weiping Li, Nan Ji, Guodong Huang

**Affiliations:** 1 Present Address: Neurosurgical Department, Shenzhen Second People’s Hospital, The First Affiliated Hospital of Shenzhen University, No.3002 Sungang Road, Futian District, Shenzhen, 518035 Guangdong Province China; 2 Neurosurgical Department, Beijing Tiantan Hospital, Capital Medical University, Beijing, 100070 China; 3 Shenzhen University Health Science Center, Shenzhen, 518000 Guangdong Province China; 4 Nephrological Department, Shenzhen Second People’s Hospital, The First Affiliated Hospital of Shenzhen University, Shenzhen, 518035 Guangdong Province China; 5 China National Clinical Research Center for Neurological Diseases, Beijing, China; 6 Advanced Innovation Center for Big Data-Based Precision Medicine, Beijing, China

**Keywords:** Platelet, Brain tumor, Craniotomy, Mortality, Non-linear

## Abstract

**Background:**

Evidence regarding the relationship between preoperative platelet and 30-day postoperative mortality of intracranial tumor patients undergoing craniotomy is still limited. Therefore, the present research was conducted to explore the link of the platelet and 30-day postoperative mortality.

**Methods:**

Electronic medical records of 18,642 adult patients undergoing craniotomy for brain tumors from 2012 to 2015 in the American College of Surgeons National Surgical Quality Improvement Program, were subject to secondary retrospective analysis. A binary logistic regression model evaluated the independent association between preoperative platelet and 30-day postoperative mortality. A generalized additive model and smooth curve fitting was conducted to explore the exact shape of the curve between them. Additionally, We also conducted sensitivity analyses to test the robustness of the results, and performed subgroup analyses.

**Results:**

Eighteen thousand sixty-three patients were included in this study analysis. Of these, 47.49% were male. The mean preoperative platelet value was (244.12 ± 76.77) × 10^9^/L. The 30-day postoperative mortality of included participants was 2.5% (452/18,063). After adjusting covariates, the results showed that preoperative platelet was positively associated with 30-day postoperative mortality (OR = 0.999, 95%CI: 0.997, 1.000). There was also a nonlinear relationship between preoperative platelet and 30-day postoperative mortality, and the inflection point of the platelet was 236. The effect sizes (OR) on the right and left sides of the inflection point were 1.002 (1.000, 1.004) and 0.993 (0.990, 0.995), respectively. And sensitive analysis demonstrated the robustness of the results. Subgroup analysis showed a stronger association between preoperative platelet and 30-day postoperative mortality in non-emergency surgery patients when preoperative platelet value is less than 235 × 10^9^/L.

**Conclusions:**

This research demonstrates a positive and non-linear relationship between preoperative platelet and 30-day postoperative mortality in U.S. adult brain tumor patients undergoing craniotomy. Preoperative platelet is strongly related to 30-day postoperative mortality when the platelet is less than 235 × 10^9^/L. Proper preoperative management of platelet and maintenance of platelet near inflection point (235) could reduce risk of 30-day postoperative mortality in these cases.

## Background

Craniotomies for brain tumor resections are common neurological procedures. However, craniotomies for brain tumors carry significant risks of adverse events, including postoperative death. Patient postoperative 30-day mortality (also called 30-day postoperative mortality), a meaningful indicator of perioperative outcome, is defined as death within 30 days after surgery. It is not only the adjustment for patient characteristics and surgeon fixed effects, but also an effective evaluation of access to safety of the anesthesia and operation [1, 2]. Previous studies reported that 30-day postoperative mortality varied from 0.95 to 8.62% [1, 3]. The 30-day postoperative mortality were 1.4–2.7% after craniotomy for intracranial tumors in children [4] and 2.46% in the craniotomy for brain tumors in adults cohort [5].

Platelets (PLT), continuously produced from megakaryocytes mainly in the bone marrow, are implicated not only in thrombosis and haemostasis, but also in other pathophysiological and physiological processes [6]. Thrombocytopenia was defined as a PLT count of less than 100 × 10^9^/L and thrombocytosis was defined as a PLT count of more than 300 × 10^9^/L [7, 8]. Accumulating studies have shown that preoperative PLT count is a prognostic indicator in all kinds of cancer surgeries [9], including colorectal cancer [10], esophageal squamous cell carcinoma [11], non-small cell lung cancer [12], gynecological tumor [13], hepatocellular carcinoma [14], head and neck squamous cell carcinoma [15], and primary gallbladder cancer [16]. Lower PLT count was associated with increase the risk of bleeding in cancer patients [17] and mortality and reoperation after craniotomy for tumor [18]. High PLT count associated with venous thromboembolism in cancer patients [19, 20]. Brain tumor patients have a 20–30% risk of venous thromboembolism, with treatment complicated by risk of cerebral hemorrhage [21]. Thus it is important to clarify the potential association between preoperative PLT level and the prognosis of patients with brain tumor.

There are limited data regarding the association of preoperative PLT levels and outcomes in patients undergoing craniotomies. Preoperative PLT-to-lymphocyte ratio (PLR) was predictive of patient survival in the glioblastomas (GBM) [22]. In addition, Dasenbrock HH, et al. maintained that preoperative moderate (a PLT count of 100,000-124,000/μL) and severe (a PLT count of 75,000-99,000/μL) thrombocytopenia were associated with mortality and reoperation after craniotomy for tumor in the National Surgical Quality Improvement Program (NSQIP) analysis [18]. Their study reported an association between preoperative thrombocytopenia and 30-day postoperative mortality in patients who underwent craniotomy for tumors. To date, no studies have explored the possibility of a nonlinear relationship or performed subgroup analyses between preoperative PLT and postoperative 30-day mortality. Thus, the present research was designed to explore the relationship between them in cross-sectional study data from a large in U.S. adult brain tumor population. This study could provide guidance for clinical practice by elucidating the quantitative relationship between preoperative PLT and 30-day postoperative mortality.

## Participants and methods

### Study design

This cross-sectional study utilized data recorded between 2012 and 2015 by the American College of Surgeons National Surgical Quality Improvement Program (ACS NSQIP) database provided in published papers [5]. Our independent variable was preoperative PLT and our dependent variable was 30-day postoperative mortality.

### Data source

Jingwen Zhang et al. published an article named “Sepsis and septic shock after craniotomy: Predicting a significant patient safety and quality outcome measure” (DOI: 10.1371/journal.pone.0235273) and originally uploaded the ACS NSQIP database from 2012 to 2015 [5] which was used for secondary analysis in our study. That original research was an open access article distributed under a Creative Commons Attribution License, which allowed unrestricted reproduction, distribution, and use in any medium, provided the original source and author are acknowledged. Therefore, this data was freely available for us to use in our secondary analyses without infringement on the authors’ rights.

### Participants

**Fig. 1:**
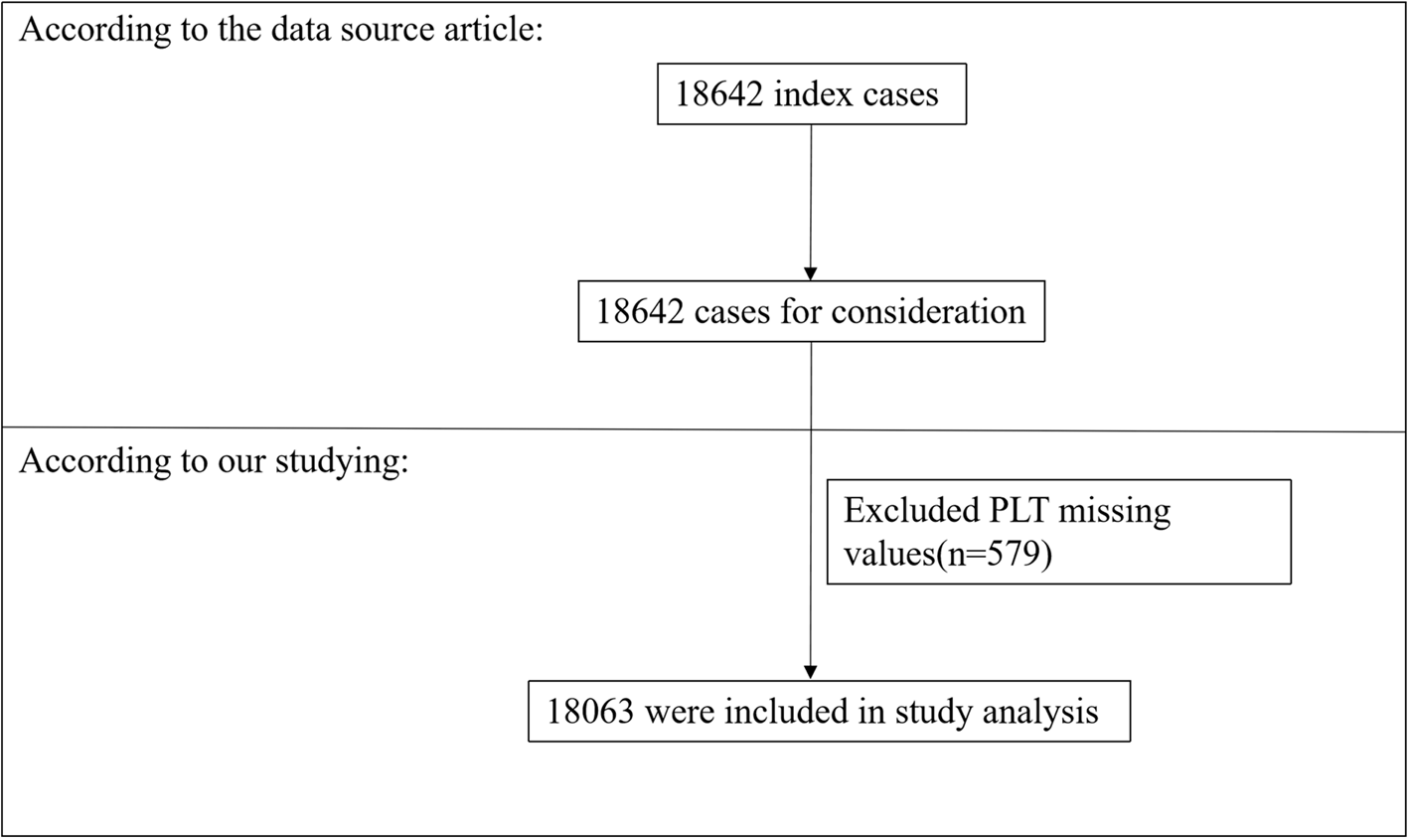
Flowchart of the study participants

The original study initially enrolled 18,642 individuals. After excluding cases with missing values of preoperative PLT (*N* = 579), 18,063 cases were included in our study analysis (as shown in Fig. 1). Because our research was based on a secondary analysis of a previously collected dataset, and the original personal information was anonymous, participants’ consent was not needed.

### Variables

Preoperative PLTs (10× 10^9^/L) were recorded as continuous variables in the original uploaded data [5].

### Thirty-day postoperative mortality

30-day postoperative mortality was defined as mortality during the first 30 days after brain tumor craniotomy [5].

### Covariates

Covariates were selected according to the previous literature and our clinical experience in our study. Height and body weight were recorded as continuous variables. Body mass index (BMI) was calculated as weight (kg) divided by height (m) squared (kg/m^2^). The original data were collected under standard conditions and processed following uniform procedures. Hence, the following variables were treated as covariates: [1] continuous variables: preoperative blood test indicators (serum sodium (Na), blood urea nitrogen (BUN), creatine (Cr), white blood cell (WBC) count, platelet (PLT) count) and BMI; and [2] categorical variables: sex (female or male), race (Asian, White, African American or Unknown race), age ranges (18–40, 41–60, 60–80, > 80 years old), dialysis, a history of diabetes (No, Yes (Noninsulin-dependent) or Yes (Insulin-dependent)), steroid use for chronic condition, smoking status, severe chronic obstructive pulmonary disease (COPD), hypertension, bleeding disorders, preoperative transfusions, preoperative systemic sepsis, and emergency case. Further details are presented in the original study [5]. In addition, a WBC count ≤10 × 10^9^/L were considered low risk, and that > 10 × 10^9^/L were considered high risk [23]. The continuous variable - WBC count was divided into dichotomous variables (low or high risk based on literature reports) for subgroup analyses.

### Statistical analysis

We stratified the participants by quartiles of preoperative PLT. For continuous outcomes, we extracted the median (interquartile range) (nonnormally distributed variables) or mean ± standard deviation (SD) (normally distributed variables); for categorical variables, we reported percentages and frequencies. We used χ^2^ tests (for categorical variables), one-way ANOVAs (for normally distributed variables), or Kruskal–Wallis H tests (for nonnormally distributed data) to test for significant differences among the different HCT groups. To examine the link between preoperative PLT and 30-day postoperative mortality, the distinct univariate and multivariate binary logistic regression models were constructed according to the Strengthening the Reporting of Observational Studies in Epidemiology (STROBE) statement guidelines, including a non-adjusted model (no covariates were adjusted), a minimally adjusted model (adjusting for age range, race, sex), and a fully adjusted model (adjusting for BMI, serum Na, BUN, Cr, WBC and HCT count, sex, race, age range, diabetes, dialysis, smoking status, severe COPD, hypertension, steroid use for chronic condition, operation time, bleeding disorders, preoperative transfusions, preoperative systemic sepsis, emergency case; covariates are presented in Table 1). Effect sizes and their 95% confidence intervals (CIs) were recorded. We adjusted the effect sizes when covariances were added to the model and the matched odds ratio was changed by 10% or more [24].

**Table 1:** Baseline demographic characteristics of the participants

PLT (quartile)	Q1 (15.00–193.00)	Q2 (194.00–235.00)	Q3 (236.00–284.00)	Q4 (285.00–988.00)	*P* value
*N* (cases)	4482	4508	4521	4552	
BMI, (Mean ± SD)	28.443 ± 6.144	28.825 ± 6.592	28.815 ± 7.034	28.751 ± 7.146	0.026
Na, (Mean ± SD)	138.848 ± 3.307	138.779 ± 3.096	138.690 ± 3.060	138.191 ± 3.237	< 0.001
Cr, (Mean ± SD)	0.918 ± 0.512	0.872 ± 0.465	0.840 ± 0.336	0.818 ± 0.439	< 0.001
WBC, median (quartile)	7.320 (5.600–10.100)	8.000 (6.200–10.900)	8.600 (6.700–11.700)	10.000 (7.600–13.500)	< 0.001
HCT, (Mean ± SD)	39.946 ± 5.203	40.765 ± 4.568	40.772 ± 4.499	39.773 ± 4.828	< 0.001
BUN, median (quartile)	16.527 (13.000–22.000)	16.000 (13.000–20.000)	16.000 (12.000–19.048)	16.000 (12.000–20.000)	< 0.001
Operation time, median (quartile)	179.000 (120.250–259.000)	183.000 (123.000–270.000	184.000 (122.000–273.000	178.000 (117.000–266.000)	0.014
Sex N (%)					< 0.001
Male	2710 (60.464%)	2337 (51.841%)	1999 (44.216%)	1532 (33.656%)	
Female	1772 (39.536%)	2171 (48.159%)	2522 (55.784%)	3020 (66.344%)	
Race N (%)					0.002
White	3264 (72.825%)	3219 (71.406%)	3215 (71.113%)	3124 (68.629%)	
Asian	120 (2.677%)	137 (3.039%)	120 (2.654%)	149 (3.273%)	
African American	275 (6.136%)	292 (6.477%)	291 (6.437%)	357 (7.843%)	
Unknown race	823 (18.362%)	860 (19.077%)	895 (19.797%)	922 (20.255%)	
Age ranges N (%)					< 0.001
18–40	586 (13.075%)	749 (16.615%)	806 (17.828%)	780 (17.135%)	
41–60	1622 (36.189%)	1841 (40.839%)	1938 (42.867%)	2098 (46.090%)	
61–80	2034 (45.382%)	1790 (39.707%)	1639 (36.253%)	1583 (34.776%)	
>81	240 (5.355%)	128 (2.839%)	138 (3.052%)	91 (1.999%)	
Diabetes *N* (%)					< 0.001
No	3867 (86.278%)	4000 (88.731%)	4032 (89.184%)	4039 (88.730%)	
Yes (Noninsulin)	368 (8.211%)	337 (7.476%)	300 (6.636%)	336 (7.381%)	
Yes (Insulin)	247 (5.511%)	171 (3.793%)	189 (4.180%)	177 (3.888%)	
Smoking status *N* (%)					< 0.001
No	3783 (84.404%)	3687 (81.788%)	3590 (79.407%)	3502 (76.933%)	
Yes	699 (15.596%)	821 (18.212%)	931 (20.593%)	1050 (23.067%)	
Severe COPD *N* (%)					< 0.001
No	4302 (95.984%)	4329 (96.029%)	4324 (95.643%)	4287 (94.178%)	
Yes	180 (4.016%)	179 (3.971%)	197 (4.357%)	265 (5.822%)	
Hypertension *N* (%)					< 0.001
No	2605 (58.121%)	2810 (62.334%)	2872 (63.526%)	2871 (63.071%)	
Yes	1877 (41.879%)	1698 (37.666%)	1649 (36.474%)	1681 (36.929%)	
Dialysis					< 0.001
No	4455 (99.398%)	4500 (99.823%)	4512 (99.801%)	4540 (99.736%)	
Yes	27 (0.602%)	8 (0.177%)	9 (0.199%)	12 (0.264%)	
Steroid use for chronic condition *N* (%)					< 0.001
No	3601 (80.344%)	3911 (86.757%)	3917 (86.640%)	3896 (85.589%)	
Yes	881 (19.656%)	597 (13.243%)	604 (13.360%)	656 (14.411%)	
Bleeding disorders *N* (%)					< 0.001
No	4283 (95.560%)	4448 (98.669%)	4459 (98.629%)	4500 (98.858%)	
Yes	199 (4.440%)	60 (1.331%)	62 (1.371%)	52 (1.142%)	
Preoperation transfusions *N* (%)					0.003
No	4457 (99.442%)	4500 (99.823%)	4512 (99.801%)	4531 (99.539%)	
Yes	25 (0.558%)	8 (0.177%)	9 (0.199%)	21 (0.461%)	
Preoperation systemic sepsis *N* (%)					< 0.001
No	4321 (96.408%)	4357 (96.650%)	4392 (97.147%)	4323 (94.969%)	
SIRS	160 (3.570%)	147 (3.261%)	127 (2.809%)	225 (4.943%)	
Septic Shock	1 (0.022%)	4 (0.089%)	2 (0.044%)	4 (0.088%)	
Emergency case *N* (%)					0.578
No	4194 (93.574%)	4215 (93.500%)	4232 (93.608%)	4232 (92.970%)	
Yes	288 (6.426%)	293 (6.500%)	289 (6.392%)	320 (7.030%)	
30 day mortality events, *N* (%)					< 0.001
No	4313 (96.229%)	4415 (97.937%)	4444 (98.297%)	4439 (97.518%)	
Yes	169 (3.771%)	93 (2.063%)	77 (1.703%)	113 (2.482%)	

The nonlinear relationship between preoperative PLT and 30-day postoperative mortality was addressed using a generalized additive model (GAM) and smooth curve fitting. If nonlinearity was detected, we calculated the inflection point using a recursive algorithm and subsequently constructed a piecewise binary logistic regression model with one piece on each side of the inflection point. The log-likelihood ratio test was employed to determine the most appropriate model for describing the association between PLT and 30-day postoperative mortality.

We performed subgroup analyses using a stratified binary logistic regression model for several subgroups: sex, age range, race, diabetes, smoking status, severe COPD, hypertension, disseminated cancer, steroid use for chronic condition, emergency case, and WBC count. We first converted the WBC count to a categorical variable based on the clinical threshold mentioned above (≤10× 10^9^/L, > 10 × 10^9^/L) [23]. Second, in addition to the stratification factor itself, we adjusted each stratification for all factors (sex, race, BMI, serum Na, BUN, Cr, WBC and HCT count, age range, diabetes, dialysis, smoking status, steroid use for chronic condition, severe COPD, preoperative transfusions, hypertension, bleeding disorders, preoperative systemic sepsis, operation time, emergency cases). Finally, we tested for interaction by employing the likelihood ratio test for models with and without interaction terms [25, 26].

Several sensitivity analyses were performed to test the robustness of our results. We converted preoperative PLT into a categorical variable according to its quartiles and calculated the P for each trend to verify the results of using PLT as a continuous variable and test the possibility of nonlinearity. We also explored the potential for unknown confounders on the relationship between preoperative PLT and 30-day postoperative mortality by calculating E-values [27]. We reported all results according to the STROBE statement guidelines.

The number of participants with missing values for Na, BUN, Cr, WBC, and HCT was 412 (2.28%), 1130 (6.26%), 367 (2.03%), 32 (0.18%), and 20 (0.11%), respectively. We replaced the missing values were by the median or mean value for statistical analysis.

Modeling was performed with the statistical software EmpowerStats (http://www.empowerstats.com, X&Y Solutions, Inc., Boston, MA) and R package (http://www.R-project.org, The R Foundation). A two-sided *P* value < 0.05 was considered statistically significant.

## Results

### Baseline characteristics of the participants

The demographic and clinical characteristics of the 18,063 included participants based on the quartiles of preoperative PLT are shown in Table 1. A total of 47.49% of the included cases were male. The age distribution proportions were 16.17% (18–40), 41.52% (41–60), 39.01% (61–80) and 3.31% (> 81). The mean preoperative PLT value was (244.12 ± 76.77) × 10^9^/L. The 30-day postoperative mortality of the included participants was 2.5% (452/18,063). We assigned included participants into subgroups using PTL quartiles: Q1 (5.00–193.00), Q2 (194.00–235.00), Q3 (236.00–284.00), and Q4 (285.00–988.00). Compared with those of participants with a lower PLT (5.00–193.00), the highest PLT (285.00–988.00) was significantly positively correlated with BMI, serum Na, Cr, WBC count, HCT count and BUN, age range, operation time, smoking status, sex, race, diabetes, severe COPD, bleeding disorders, hypertension, dialysis, steroid use for chronic condition, preoperative transfusions, preoperative systemic sepsis, 30 day mortality events (all *P* values < 0.05) except emergency case (P values > 0.05). Although these baseline indicators of preoperative blood tests (such as serum HCT, BUN and Na) were statistically significant due to the large sample size, they were not clinically significant.

### The results of the univariate analysis using a binary logistic regression model

The univariate analysis indicated that patients that were female, 41–60 years old, 61–80 years old, > 81 years old, had diabetes (noninsulin-dependent), diabetes (insulin-dependent), severe COPD, hypertension, dialysis, steroid use for chronic condition, bleeding disorders, levels of Na, Cr, WBC, PLT, HCT, BUN, preoperative transfusions, preoperative SIRS, preoperative septic shock, operation time, and emergency case were positively associated with 30-day postoperative mortality. In contrast, patients that were Asian, African American, of unknown race, BMI and those that smoked were negatively associated with 30-day postoperative mortality (Table 2 provides more details).

**Table 2:** The results of the univariate analysis

	Statistics	OR	95% CI	*P* value
Sex, N (%)				
Male	8578 (47.489%)	Ref.		
Female	9485 (52.511%)	0.643	(0.532, 0.777)	< 0.00001
Race, N (%)				
White	12,822 (70.985%)	Ref.		
Asian	526 (2.912%)	0.767	(0.406, 1.448)	0.41330
African American	1215 (6.726%)	0.899	(0.604, 1.338)	0.60128
Unknown race	3500 (19.377%)	1.152	(0.916, 1.448)	0.22561
Age range, N (%)				
18–40	2921 (16.171%)	Ref.		
41–60	7499 (41.516%)	2.780	(1.737, 4.448)	0.00002
61–80	7046 (39.008%)	5.005	(3.164, 7.916)	< 0.00001
>81	597 (3.305%)	15.014	(8.938, 25.222)	< 0.00001
BMI	28.709 ± 6.743	0.986	(0.972, 1.001)	0.07702
Diabetes, N (%)				
No	15,938 (88.236%)	Ref.		
Yes (Noninsulin-dependent)	1341 (7.424%)	1.528	(1.119, 2.088)	0.00770
Yes (Insulin-dependent)	784 (4.340%)	2.558	(1.855, 3.529)	< 0.00001
Smoking status, N (%)				
No	14,562 (80.618%)	Ref.		
Yes	3501 (19.382%)	1.125	(0.895, 1.414)	0.31210
Severe COPD, N (%)				
No	17,242 (95.455%)	Ref.		
Yes	821 (4.545%)	2.524	(1.851, 3.444)	< 0.00001
Hypertension, N (%)				
No	11,158 (61.773%)	Ref.		
Yes	6905 (38.227%)	2.256	(1.867, 2.725)	< 0.00001
Dialysis, N (%)				
No	18,007 (99.690%)	Ref.		
Yes	56 (0.310%)	6.593	(3.101, 14.018)	< 0.00001
Steroid use for chronic condition N (%)				
No	15,325 (84.842%)	Ref.		
Yes	2738 (15.158%)	2.349	(1.909, 2.890)	< 0.00001
Bleeding disorders N (%)				
No	17,690 (97.935%)	Ref.		
Yes	373 (2.065%)	2.263	(1.428, 3.587)	0.00051
Na	138.626 ± 3.187	0.911	(0.887, 0.936)	< 0.00001
Cr	0.862 ± 0.444	1.246	(1.118, 1.389)	0.00007
WBC	9.507 ± 4.474	1.073	(1.056, 1.090)	< 0.00001
PLT	244.122 ± 76.773	0.998	(0.997, 0.999)	0.00154
HCT	40.313 ± 4.803	0.922	(0.907, 0.938)	< 0.00001
BUN	17.324 ± 8.076	1.043	(1.035, 1.050)	< 0.00001
Preoperative transfusions N (%)				
No	18,000 (99.651%)	Ref.		
Yes	63 (0.349%)	5.751	(2.723, 12.147)	< 0.00001
preoperative systemic sepsis N (%)				
No	17,393 (96.291%)	Ref.		
SIRS	659 (3.648%)	2.599	(1.854, 3.644)	< 0.00001
Septic Shock	11 (0.061%)	9.182	(1.978, 42.630)	0.00465
Operation time	211.344 ± 131.693	0.998	(0.998, 0.999)	0.00011
Emergency case N (%)				
No	16,873 (93.412%)	Ref.		
Yes	1190 (6.588%)	2.844	(2.198, 3.680)	< 0.00001

### Multivariate analyses using the binary logistic regression model

The authors constructed three models using the binary logistic regression model to investigate the association between preoperative PLT and 30-day postoperative mortality. In the nonadjusted model, an increase of 1 unit of PLT was related to a 0.2% reduce in 30-day postoperative mortality (OR = 0.998, 95% CI 0.997 to 0.999). The results were statistically significant. In the minimally-adjusted model, when the authors adjusted for sex, race and age ranges, each additional unit of PLT increase could lead to elevated the 30-day postoperative mortality by 0.1% (OR = 0.999, 95% CI 0.998 to 1.000). In the fully adjusted model, each additional PLT unit was accompanied by a 0.1% increase in postoperative 30-day mortality (OR = 0.999, 95% CI 0.997 to 1.000). The results were statistically significant. The distribution of confidence intervals indicated that the link between the preoperative PLT and 30-day postoperative mortality obtained by the model was reliable (Table 3).

**Table 3:** Multivariate analysis on the association between preoperative PLT and 30-day postoperative mortality

Exposure	Model 1 (OR, 95% CI,P)	Model 2 (OR, 95% CI,P)	Model 3 (OR, 95% CI,P)
PLT	0.998 (0.997, 0.999) 0.00154	0.999 (0.998, 1.000) 0.22630	0.999 (0.997, 1.000) 0.03982
PLT (quartile)			
Q1			
Q2	0.538 (0.416, 0.695) < 0.00001	0.622 (0.480, 0.807) 0.00034	0.664 (0.503, 0.877) 0.00395
Q3	0.442 (0.337, 0.581) < 0.00001	0.535 (0.406, 0.706) < 0.00001	0.558 (0.416, 0.749) 0.00010
Q4	0.650 (0.510, 0.827) 0.00047	0.851 (0.662, 1.094) 0.20793	0.718 (0.545, 0.945) 0.01820
P for trend	0.00006	0.05543	0.00509

Model 1 (nonadjusted model): not adjust any covariate

Model 2 (minimally-adjusted model): adjusted age ranges, sex, race

Model 3 (fully-adjusted model): adjusted BMI, sex, age range, race, dialysis, diabetes, smoking status, bleeding disorders,severe COPD, hypertension, preoperative transfusions, steroid use for chronic condition, preoperative systemic sepsis, operation time, emergency case, serum Na, BUN, Cr, WBC count, and HCT count

*OR* odds ratios; *CI* confidence; *BMI* body mass index; *COPD* chronic obstructive pulmonary disease

### Sensitivity analyses

A series of sensitivity analyses were performed to verify the robustness of our findings. We first converted preoperative PLT from a continuous variable to a categorical variable (dividing into groups according to quartiles) and then replaced previous PLT variable in the model with the categorical-transformed PLT. After preoperative PLT was transformed into a categorical variable, the trend of the effect sizes in different groups was equidistant, and the P for the trend was consistent with the result when PLT was a continuous variable.

In addition, a GAM was used to insert the continuity covariate into the equation as a curve. The E-value was also computed to assess the sensitivity to unmeasured confounders and the E-value was 1.03, which was greater than the relative risk of unmeasured confounders influencing the relationship between preoperative PLT and 30-day postoperative mortality, suggesting that unknown or unmeasured confounders had little effect on the relationship.

### The nonlinearity addressed by the generalized additive model

**Fig. 2:**
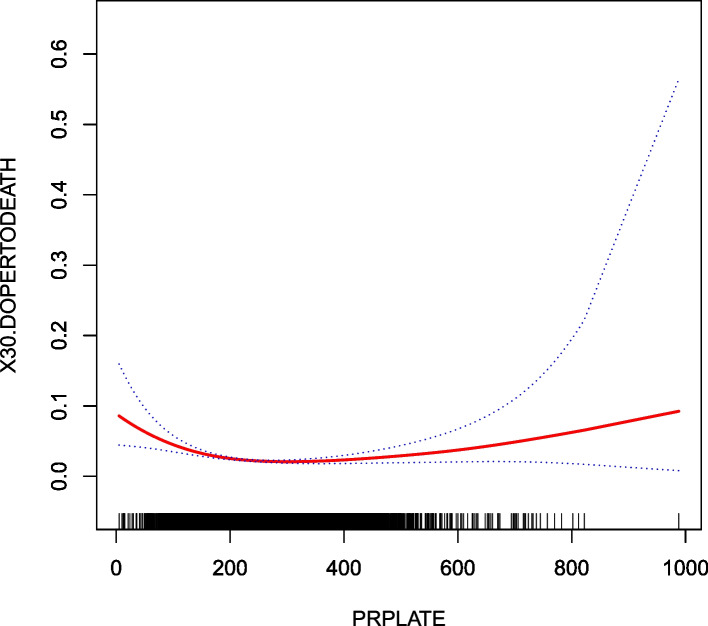
The nonlinear relationship between preoperative PLT and 30-day postoperative mortality

Through the GAM and smooth curve fitting, we observed that the association between preoperative PLT and 30-day postoperative mortality was nonlinear (Fig. 2). Therefore, we fit the data to a piecewise binary logistic regression model that allowed two different slopes. Data were also fitted by a standard binary logistic regression model based on the sensitivity analysis, and the best appropriate model was selected through the log-likelihood ratio test (Table 4). The P for the log-likelihood ratio test was < 0.05 in this study. Therefore, a piecewise model was used to fit the link between preoperative PLT and 30-day postoperative mortality. With a recursive algorithm, we first obtained an inflection point of 236 and then calculated the effect sizes and CIs to the left and right of the inflection point with the piecewise binary logistic regression model. On the left side of the inflection point, the effect size was 0.993, and the 95% CI was from 0.990 to 0.995. On the right side of the inflection point, the effect size was 1.002, and the 95% CI was from 1.000 to 1.004.

**Table 4:** The results of the standard linear and two-piecewise linear regression model

	Postoperative 30-day mortality (OR, 95% CI, *P* value)
Fitting model by standard linear regression	0.999 (0.997, 1.000) 0.0398
Fitting model by two-piecewise linear regression	
Infection point	236
≤236	0.993 (0.990, 0.995) < 0.0001
>236	1.002 (1.000, 1.004) 0.0179
*P* value for log likelihood ratio test	< 0.001

The model adjusted for BMI, sex, race, age range, bleeding disorders, diabetes, dialysis, smoking status, severe COPD, preoperative transfusions, hypertension, steroid use for chronic condition, preoperative systemic sepsis, operation time, emergency case, serum Na, BUN, Cr, WBC count, and HCT count

*Ref* reference; *OR* odds ratios; *CI* confidence

### Subgroup analyses

We performed subgroup analyses to consider other factors that could impact the relationship between preoperative PLT and 30-day postoperative mortality. We used sex, age range, diabetes, bleeding disorders, smoking status, pre-operation transfusions, severe COPD, emergency case, hypertension, steroid use for chronic condition and WBC count as stratification variables to detect the trend of effect sizes. Table 5 showed that there were no significant differences in the relationship in different sex, age range, diabetes, smoking status, severe COPD, hypertension, steroid use for chronic condition, bleeding disorders, pre-operation transfusions, and WBC groups (P for interaction > 0.05); only emergency case modified the relationship between preoperative PLT and 30-day postoperative mortality (P for interaction < 0.05). The stronger association was observed in non-emergency surgery patients (OR = 0.998, 95% CI: 0.996–0.999), and the weaker association was observed in emergency surgery patients (OR = 1.002, 95% CI: 0.999–1.005).

**Table 5:** Results of interaction and subgroup analyses

Characteristic	No of participants	OR (95% CI)	P for interaction
Sex			0.7065
Male	8578	0.999 (0.997, 1.000)	
Female	9485	0.998 (0.996, 1.000)	
Age ranges			0.1822
18–40	2921	0.996 (0.989, 1.004)	
41–60	7499	0.997 (0.995, 0.999)	
61–80	7046	1.000 (0.998, 1.002)	
> 81	597	0.997 (0.993, 1.002)	
Diabetes			0.8235
No	15,938	0.999 (0.997, 1.000)	
Yes (Noninsulin)	1341	0.999 (0.995, 1.003)	
Yes (Insulin)	784	0.997 (0.992, 1.002)	
Smoking status			0.7369
No	14,562	0.998 (0.997, 1.000)	
Yes	3501	0.999 (0.997, 1.001)	
Severe COPD			0.6776
No	17,242	0.999 (0.997, 1.000)	
Yes	821	0.998 (0.994, 1.002)	
Hypertension			0.5515
No	11,158	0.998 (0.996, 1.000)	
Yes	6905	0.999 (0.997, 1.001)	
Steroid use for chronic condition			0.9858
No	15,325	0.999 (0.997, 1.000)	
Yes	2738	0.999 (0.996, 1.001)	
Bleeding disorders			0.2669
No	17,690	0.999 (0.998, 1.000)	
Yes	373	0.994 (0.984, 1.003)	
Pre-operation transfusions			0.2426
No	18,000	0.999 (0.997, 1.000)	
Yes	63	1.003 (0.996, 1.011)	
Emergency case			0.0110
No	16,873	0.998 (0.996, 0.999)	
Yes	1190	1.002 (0.999, 1.005)	
WBC			0.2615
WBC ≤ 10	6459	0.998 (0.996, 1.000)	
WBC > 10	11,604	1.000 (0.997, 1.002)	

### Emergency case differences in the nonlinear relationship

**Fig. 3:**
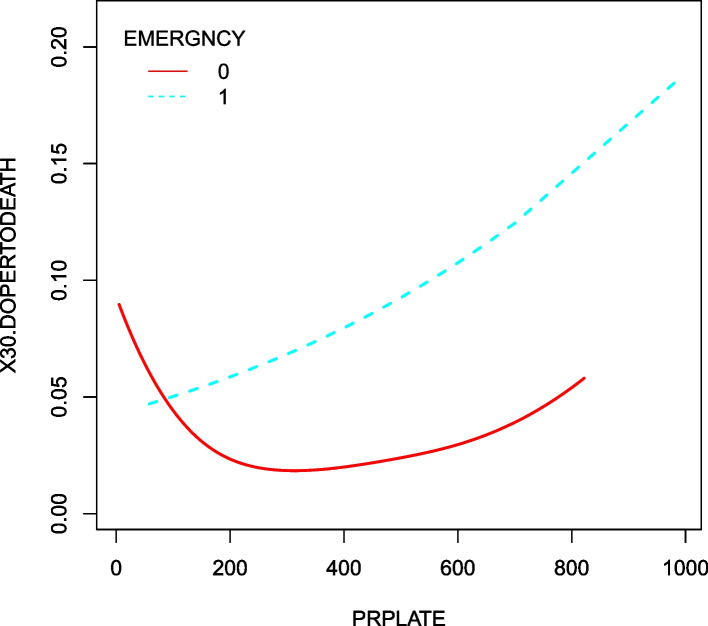
Emergency case differences in the effect of preoperative PLT on 30-day postoperative mortality

Based on the above results, we further explored the effect of emergency case on the relationship between preoperative PLT and 30-day postoperative mortality. The nonlinearity was addressed by a GAM. We found a specific nonlinear relationship between preoperative PLT and 30-day postoperative mortality both in non-emergency and emergency surgery patients (Fig. 3). As shown in Table 6, a recursive algorithm found that two inflection points were 235 and 195, respectively. Curve fitting and threshold effect analysis revealed that for non-emergency surgery patients with preoperative PLT value ≤235, a one unit decrease in PLT was related to a 0.8% increase in risk of 30-day postoperative mortality (OR = 0.992, 95%CI (0.989, 0.995); for non-emergency surgery patients with preoperative PLT value > 235, no significant association was found between PLT and 30-day postoperative mortality. No significant association was also found between PLT and 30-day postoperative mortality in emergency surgery patients (*P* value > 0.05).

**Table 6:** The results of the piecewise linear regression

Emergency case	No 30-day mortality (OR, 95% CI, *P* value)		Yes 30-day mortality (OR, 95% CI, *P* value)
Infection point of PLT	235		195
≤235	0.992 (0.989, 0.995) < 0.0001	**≤** 195	0.995 (0.983, 1.007) 0.4273
>235	1.001 (1.000, 1.003) 0.1413	**>** 195	1.003 (1.000, 1.006) 0.0389
*P* value from the log likelihood ratio test	< 0.001		0.250

The model adjusted for BMI, sex, race, age range, bleeding disorders, diabetes, dialysis, smoking status, severe COPD, preoperative transfusions, hypertension, steroid use for chronic condition, preoperative systemic sepsis, operation time, serum Na, BUN, Cr, WBC count, and HCT count

*OR* odds ratios; *CI* confidence

## Discussion

In this study, we used the ACS NSQIP (2012–2015) database to explore whether there was an association between preoperative PLT and 30-day postoperative mortality. We found that the decrease of the preoperative PLT was related to a significantly increased 30-day postoperative mortality. In addition, a threshold effect curve was found as well, and different relationships of preoperative PLT on 30-day postoperative mortality were detected on both sides of the inflection point. In addition, emergency case was found as the potential effect modifiers to modify the relationship between preoperative PLT and 30-day postoperative mortality.

Through a literature search, we found only two pieces of literature currently investigating the relationship between preoperative PLT and brain tumor patients’ outcomes [18, 22]. One study included 14,852 patients who underwent craniotomy for brain tumor of the prospective NSQIP registry (2007–2014). The study found that moderate (100,000-124,000/μL) and severe (75,000-99,000/μL) thrombocytopenia were associated with 30-day postoperative mortality and reoperation after craniotomy for brain tumor [18]. The present data-analysis study also used the NSQIP database for analysis and we agreed with their conclusion that lower platelets were associated with 30-day postoperative mortality. Surgical bleeding can lead to diffuse bleeding as platelets and coagulation factors are already reduced [28]. We believe that patients with low platelets in general have further loss of platelets and clotting factors during surgery, which may increase the risk of bleeding and account for the difference in prognosis for these patients. Unlike their study, our study used a two-piecewise linear regression model to clarify a non-linear relationship between the preoperative PLT and the 30-day postoperative mortality. The inflection point was 236 after adjusting for confounders and emergency case could modified the relationship between them (P for interaction < 0.05). Curve fitting and threshold effect analysis revealed that for non-emergency surgery patients with preoperative PLT ≤ 235, a one unit decrease in PLT was related to a 0.8% increase in risk of 30-day postoperative mortality (OR = 0.992, 95%CI (0.989, 0.995); for non-emergency surgery patients with preoperative PLT > 235, the effect of a one unit increase in preoperative PLT was related to a 0.1% increase in risk of 30-day postoperative mortality (OR = 1.001, 95%CI (1.000, 1.003). The inflection point provides firstly evidence for the preoperative PLT management in the U.S. population. Therefore, this analysis has excellent clinical value. Another study in China included 141 primary GBM and 25 secondary GBM. The results suggested that PLR was predictive of patient survival in the GBM, primary GBM, and IDH-wild type glioblastoma (IDH-wt GBM) groups. The mechanism underlying the prognostic role of PLR remains unclear in GBM. Future large samples and further mechanism studies are needed.

### Strengths and limitations

Importantly, to the best of our knowledge, this is the largest retrospective study exploring the relationship between preoperative PLT and 30-day postoperative mortality, and this large sample size ensures sufficient statistical power. Compared to prior studies, the study of nonlinear addressing is a considerable advance. The study was observational and is susceptible to potential confounding. Strict statistical adjustments were utilize to minimize residual confounds. We used a GAM and smooth curve fitting (penalized spline method) to explore the nonlinear relationship. Thus, this study had greater clinical value, which previous studies did not explore. We performed subgroup analyses, and also conducted sensitivity analyses to ensure the robustness of our results. In addition, study of effect size to help use these data in future studies.

This retrospective analysis has some limitations. Because this was a secondary analysis of previously published data, we cannot rule out some unmeasured and/or residual confounding factors that may influence the estimated relationship (e.g., platelet function [29, 30], socioeconomic factors). However, we calculated the E-value to quantify the potential impact of unmeasured confounders. Besides, due to the content limitations of the uploaded database, we could not investigate the relationship between preoperative PLT and long-term outcomes. There are many causes of death after craniotomy for brain tumors. Animal experiments can be used for exploring the direct relationship. Despite these limitations, our study was based on data from a large group of U.S. adult patients in approximately 400 community hospitals and academic. As such, our conclusions postulated remain highly plausible.

## Conclusions

This research demonstrates a positive and nonlinear relationship between preoperative PLT and 30-day postoperative mortality in adult patients who underwent craniotomy for tumors. There was a threshold effect between the preoperative PLT and 30-day postoperative mortality. When the preoperative PLT counts was lower than 235 < 10× 10^9^/L, there was a significant positive association with 30-day postoperative mortality in non-emergency surgery patients. This result is expected to provide a reference for clinicians for preoperative PLT. Proper preoperative management of PLT and maintenance of PLT near inflection point (235) could reduce 30-day postoperative mortality in these cases. Therefore, abnormal preoperative PLT can help identify high-risk groups for postoperative 30-day mortality in U.S. adult patients who undergo craniotomy for tumors, which will allow clinicians to plan and initiate appropriate management strategies.

## Data Availability

The raw data can be downloaded from (https://journals.plos.org/plosone/article?id=10.1371/journal.pone.0235273, S1 Data).

## Abbreviations

PLT: platelet
ACS NSQIP: American College of Surgeons National Surgical Quality Improvement Program
PLR: PLT-to-lymphocyte ratio
GBM: Glioblastoma multiforme
CI: Confidence interval
OR: Odds ratio
SD: Standard deviation
STROBE: Strengthening the Reporting of Observational Studies in Epidemiology
GAM: Generalized additive model
BMI: Body mass index
COPD: Chronic obstructive pulmonary disease
Na: serum sodium
BUN: Blood urea nitrogen
Cr: creatine
WBC: White blood cell
HCT: Hematocrit

## References

[1] Kato H, Jena AB, Tsugawa Y (2020). Patient mortality after surgery on the surgeon's birthday: observational study. BMJ..

[2] Watters DA, Hollands MJ, Gruen RL, Maoate K, Perndt H, McDougall RJ (2015). Perioperative mortality rate (POMR): a global indicator of access to safe surgery and anaesthesia. World J Surg.

[3] Williams M, Treasure P, Greenberg D, Brodbelt A, Collins P (2016). Surgeon volume and 30 day mortality for brain tumours in England. Br J Cancer.

[4] Hankinson TC, Dudley RW, Torok MR, Patibandla MR, Dorris K, Poonia S (2016). Short-term mortality following surgical procedures for the diagnosis of pediatric brain tumors: outcome analysis in 5533 children from SEER, 2004-2011. J Neurosurg Pediatr.

[5] Zhang J, Li YI, Pieters TA, Towner J, Li KZ, Al-Dhahir MA (2020). Sepsis and septic shock after craniotomy: predicting a significant patient safety and quality outcome measure. PLoS One.

[6] van der Meijden P, Heemskerk J (2019). Platelet biology and functions: new concepts and clinical perspectives. Nat Rev Cardiol.

[7] Liao D, Zhou F, Luo L, Xu M, Wang H, Xia J (2020). Haematological characteristics and risk factors in the classification and prognosis evaluation of COVID-19: a retrospective cohort study. Lancet Haematol.

[8] Cheng YQ, Wang K, Zhang XP, Wei XB, Jiang YB, Hu YR (2019). Thrombocytopenia: a prognostic factor for hepatocellular carcinoma patients with portal vein tumor thrombus after hepatectomy. J Gastroenterol Hepatol.

[9] Rachidi S, Li H, Wallace K, Li Z, Balch C, Lautenschlaeger T (2020). Preoperative platelet counts and postoperative outcomes in cancer surgery: a multicenter, retrospective cohort study. Platelets..

[10] Wan S, Lai Y, Myers RE, Li B, Hyslop T, London J (2013). Preoperative platelet count associates with survival and distant metastasis in surgically resected colorectal cancer patients. J Gastrointest Cancer.

[11] Feng JF, Huang Y, Lu WS, Chen QX (2013). Preoperative platelet count in esophageal squamous cell carcinoma: is it a prognostic factor?. Langenbeck's Arch Surg.

[12] Liu HB, Gu XL, Ma XQ, Lv TF, Wu Y, Xiao YY (2013). Preoperative platelet count in predicting lymph node metastasis and prognosis in patients with non-small cell lung cancer. Neoplasma..

[13] Yang W, Chen YY, Bi C, Shu KY, Ye ML, Li FF (2020). Predictive and prognostic values of preoperative platelet parameters in patients with gynecological tumors. J Clin Lab Anal.

[14] Li SP, Cao D, He JH, Lou MG, Tu XX, Li Y (2022). High platelet count predicts poor prognosis in HCC patients undergoing TACE: a propensity score-matched analysis. Expert Rev Gastroenterol Hepatol.

[15] Takenaka Y, Oya R, Kitamiura T, Ashida N, Shimizu K, Takemura K (2018). Platelet count and platelet-lymphocyte ratio as prognostic markers for head and neck squamous cell carcinoma: Meta-analysis. Head Neck.

[16] Wang RT, Zhang LQ, Mu YP, Li JB, Xu XS, Pang Q (2015). Prognostic significance of preoperative platelet count in patients with gallbladder cancer. World J Gastroenterol.

[17] Vinholt PJ (2019). The role of platelets in bleeding in patients with thrombocytopenia and hematological disease. Clin Chem Lab Med.

[18] Dasenbrock HH, Devine CA, Liu KX, Gormley WB, Claus EB, Smith TR (2016). Thrombocytopenia and craniotomy for tumor: a National Surgical Quality Improvement Program analysis. Cancer-Am Cancer Soc.

[19] Simanek R, Vormittag R, Ay C, Alguel G, Dunkler D, Schwarzinger I (2010). High platelet count associated with venous thromboembolism in cancer patients: results from the Vienna Cancer and thrombosis study (CATS). J Thromb Haemost.

[20] Khorana AA, Mackman N, Falanga A, Pabinger I, Noble S, Ageno W (2022). Cancer-associated venous thromboembolism. Nat Rev Dis Primers.

[21] Perry JR (2010). Anticoagulation of malignant glioma patients in the era of novel antiangiogenic agents. Curr Opin Neurol.

[22] Wang PF, Song HW, Cai HQ, Kong LW, Yao K, Jiang T (2017). Preoperative inflammation markers and IDH mutation status predict glioblastoma patient survival. Oncotarget..

[23] Sun J, Lou Y, Zhu J, Shen H, Zhou ZL (2020). Hypertriglyceridemia in newly diagnosed acute promyelocytic leukemia. *Front*. Oncol..

[24] Vandenbroucke JP, von Elm E, Altman DG, Gotzsche PC, Mulrow CD, Pocock SJ (2007). Strengthening the reporting of observational studies in epidemiology (STROBE): explanation and elaboration. PLoS Med.

[25] Keidel D, Anto JM, Basagana X, Bono R, Burte E, Carsin AE, et al. The role of socioeconomic status in the association of lung function and air pollution-a pooled analysis of three adult ESCAPE cohorts. Int J Environ Res Public Health. 2019:16. 10.3390/ijerph16111901. 10.3390/ijerph16111901 PMC6603717 31146441

[26] Mullee A, Romaguera D, Pearson-Stuttard J, Viallon V, Stepien M, Freisling H (2019). Association between soft drink consumption and mortality in 10 european countries. JAMA Intern Med.

[27] Haneuse S, VanderWeele TJ, Arterburn D (2019). Using the E-value to assess the potential effect of unmeasured confounding in observational studies. JAMA..

[28] Hartmann M, Szalai C, Saner FH (2016). Hemostasis in liver transplantation: pathophysiology, monitoring, and treatment. World J Gastroenterol.

[29] Ghosal S, Trivedi J, Barlowe D, Zhao L, Ji X, Slaughter MS (2020). Preoperative functional platelet number is inversely associated with 30-day mortality after cardiac surgery: a retrospective cohort study. Semin Cardiothorac Vasc Anesth.

[30] Grove EL, Hossain R, Storey RF (2013). Platelet function testing and prediction of procedural bleeding risk. Thromb Haemost.

